# *In vitro* carbapenem activity in *Neisseria gonorrhoeae*

**DOI:** 10.1093/jacamr/dlag201

**Published:** 2026-09-03

**Authors:** Julie Brousseau, Aymeric Braille, Mary Mainardis, Fabienne Meunier, Manel Mérimèche, François Caméléna, Béatrice Berçot

**Affiliations:** Bacteriology Unit, Saint Louis-Lariboisière University Hospitals, APHP, Paris, France; Associated Laboratory for Gonococci, French National Reference Centre for Bacterial STI, Paris, France; IAME, INSERM1137, PrevIST Team, Paris Cité University, Paris, France; Bacteriology Unit, Saint Louis-Lariboisière University Hospitals, APHP, Paris, France; Associated Laboratory for Gonococci, French National Reference Centre for Bacterial STI, Paris, France; Bacteriology Unit, Saint Louis-Lariboisière University Hospitals, APHP, Paris, France; Associated Laboratory for Gonococci, French National Reference Centre for Bacterial STI, Paris, France; Bacteriology Unit, Saint Louis-Lariboisière University Hospitals, APHP, Paris, France; Associated Laboratory for Gonococci, French National Reference Centre for Bacterial STI, Paris, France; Bacteriology Unit, Saint Louis-Lariboisière University Hospitals, APHP, Paris, France; Associated Laboratory for Gonococci, French National Reference Centre for Bacterial STI, Paris, France; IAME, INSERM1137, PrevIST Team, Paris Cité University, Paris, France; Bacteriology Unit, Saint Louis-Lariboisière University Hospitals, APHP, Paris, France; Associated Laboratory for Gonococci, French National Reference Centre for Bacterial STI, Paris, France; IAME, INSERM1137, PrevIST Team, Paris Cité University, Paris, France; Bacteriology Unit, Saint Louis-Lariboisière University Hospitals, APHP, Paris, France; Associated Laboratory for Gonococci, French National Reference Centre for Bacterial STI, Paris, France; IAME, INSERM1137, PrevIST Team, Paris Cité University, Paris, France

## Abstract

**Objective:**

*Neisseria gonorrhoeae* is a common bacterium responsible for sexually transmitted infections. The WHO recommends the use of 1 g ceftriaxone to treat gonococcal infections. However, ceftriaxone-resistant strains are emerging, and carbapenems may be the last remaining treatment option.

**Methods:**

We compared MICs for ceftriaxone, ertapenem, imipenem and meropenem in a collection of 81 clinical isolates and nine WHO reference isolates with various *penA* alleles. This previously characterized collection included nine ceftriaxone-resistant isolates. It was selected to include 42 different *penA* alleles (27 non-mosaic and 15 mosaic), 26 TEM penicillinase-producing isolates, five MDR and four XDR isolates.

**Results:**

The MICs of ceftriaxone, ertapenem, imipenem and meropenem ranged from <0.002 to 2 mg/L, <0.002 to 0.064 mg/L, 0.008 to 1 mg/L and 0.002 to 0.125 mg/L, respectively. In ceftriaxone-resistant isolates, the MIC_50_ of ertapenem, imipenem and meropenem were 0.012 mg/L (0.004–0.032 mg/L), 0.75 mg/L (0.125–1 mg/L) and 0.032 mg/L (0.016–0.064 mg/L), respectively. For all antibiotics, MICs were significantly higher in isolates with a mosaic *penA* allele (*P*-value <0.001). No significant difference in carbapenem MICs was found between isolates with and without a TEM beta-lactamase.

**Conclusions:**

Carbapenem MICs remain low in *N. gonorrhoeae* isolates but are higher in those with mosaic *penA* alleles than in those with non-mosaic *penA* alleles. MICs for ertapenem are lower than those for other carbapenems, supporting its use for the treatment of infections with ceftriaxone-resistant *N. gonorrhoeae*. However, the establishment of breakpoints for carbapenem resistance in *N. gonorrhoeae* is required.

## Introduction

*Neisseria gonorrhoeae* is the second most common bacterium responsible for sexually transmitted infections, with an estimated 82.4 million cases of gonorrhoea worldwide in adults aged between 15 and 49 years in 2020.^1^ Recent European data show an increase in gonorrhoea cases among men who have sex with men (MSM) and young heterosexual men and women after the COVID-19 pandemic.^2^ In parallel, antimicrobial resistance has increased, and the WHO has classified *N. gonorrhoeae* resistant to ceftriaxone (MDR), the first line of treatment, as a matter of priority in its efforts to enhance antimicrobial resistance surveillance.^3^ However, extremely resistant (XDR) isolates of *N. gonorrhoeae*, defined as isolates resistant to all lines of treatment except aminoglycosides (ceftriaxone, high-dose azithromycin, tetracycline and ciprofloxacin), are emerging in Southeast Asia,^4^ with sporadic cases also reported in Europe.^5,6^ The emergence of this resistance to third- generation cephalosporins is mostly due to the propagation of the FC428 clone, first described in Asia. This clone harbours a mosaic allele of the *penA* gene, *penA*-60.001, encoding penicillin-binding protein 2, the principal target of beta-lactams.^7^ The emergence of this resistance led the WHO to update its treatment recommendations for all countries worldwide, including those in Asia, with the use of a higher dose of ceftriaxone (1 g) in monotherapy now recommended.^8^ This high dose has been shown to kill the bacterium in several clinical cases.^5^ Unfortunately, in severe clinical presentations or in cases of extragenital locations of the bacterium, such as the throat in particular, treatment with high-dose ceftriaxone may be unsuccessful, and an alternative treatment is therefore required.^9^

In this context, carbapenems may be the last treatment option remaining, particularly in the case of treatment failure on ceftriaxone. In particular, European guidelines recommend the use of ertapenem as an alternative treatment for oropharyngeal infection with ceftriaxone-resistant strains.^10^

In this study, we evaluated and compared the MICs of carbapenems in a collection of *N. gonorrhoeae* isolates harbouring various *penA* alleles.

## Methods

We included 81 *N. gonorrhoeae* isolates, nine of which were ceftriaxone resistant, from the collection of the French National Reference Centre for sexually transmitted bacterial infections at Saint-Louis Hospital in Paris, together with nine WHO reference strains, in this study. Nine of the isolates were wild-type, 72 were non-WT non-MDR isolates, five were MDR isolates, and four were XDR isolates. The isolates selected belonged to 46 different sequence types and had 42 different *penA* alleles: 27 non-mosaic alleles and 15 mosaic alleles. These isolates had previously been characterized phenotypically and genotypically by whole-genome sequencing on an Illumina MiSeq (Illumina^®^) system (300 cycles, 2 × 150 bp).^5^ Bioinformatic analyses were performed, as previously described.^11^ The MICs of ertapenem, imipenem and meropenem were determined with concentration gradient strips (Etest, BioMérieux^®^) on PVX agar plate (BioMérieux^®^) and compared with previously determined MICs for ceftriaxone, which had also been determined using the Etest method on PVX agar plate. Twenty-six of the *N. gonorrhoeae* isolates harboured the *bla*_TEM_ gene. The characteristics of the isolates are presented in Supplementary Table S1 (available as Supplementary data at *JAC-AMR* Online). No interpretative criteria have been provided by EUCAST or the Clinical and Laboratory Standards Institute (CLSI) for carbapenem susceptibility breakpoints for *N. gonorrhoeae*. Wilcoxon tests and non-parametric Spearman’s correlation tests confirmed using the Bonferroni correction to account for multiple comparisons were used for statistical analysis.

MICs of the WHO A/C/D/G/K/P/X/Y/Z *N. gonorrhoeae* isolates were also determined by dilution on antibiotic-containing agar {GC agar base medium (Difco) supplemented with Kellogg’s supplements [supplement I (thiamine 2 µg/mL in D-(+)-glucose 0.45%), supplement II (Fe(NO_3_)3 9H_2_O 12 mM) and supplement III (L-glutamine 0.01%)]}. The tests were performed in duplicate and triplicate for ertapenem, with ceftriaxone included as a control. The essential agreement (±1 dilution) or agreement within (±2 or 3 dilutions) between the Etest (BioMérieux^®^) and agar dilution was described in a Supplementary Tables S2 and S3 with methods.

## Results

The MICs of ertapenem, imipenem and meropenem ranged from <0.002 to 0.064 mg/L, 0.008 to 1 mg/L and 0.002 to 0.125 mg/L, respectively. The MIC_50_ were 0.004 mg/L, 0.125 mg/L and 0.008 mg/L, respectively. The MICs of imipenem were higher than those of the other carbapenems and ceftriaxone (*P* < 0.001). By contrast, the MICs of ertapenem were very low, with a leftward shift in distribution relative to meropenem and imipenem (*P* < 0.001).

**Figure 1.**
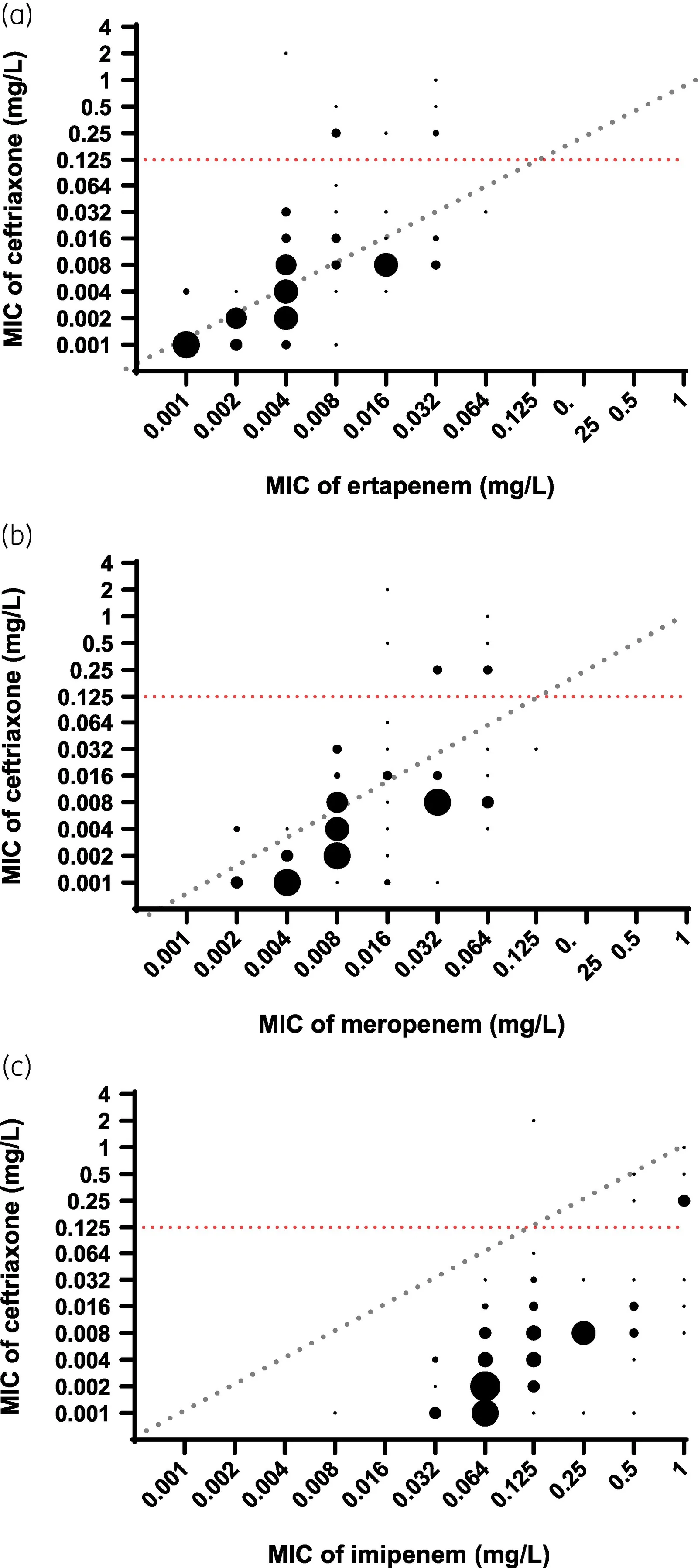
Distribution of isolates according to the MICs (in mg/L) of ceftriaxone and ertapenem (a), meropenem (b) and imipenem (c). The horizontal dotted line represents the breakpoint for ceftriaxone susceptibility, and the angled dotted line indicates equal values for the MICs of carbapenem and ceftriaxone. Dot size is proportional to the number of isolates.

The MICs of ertapenem and meropenem were similar to those of ceftriaxone in ceftriaxone-susceptible isolates, and they remained ≤0.064 mg/L and ≤0.125 mg/L, respectively, in ceftriaxone-resistant isolates (ceftriaxone MIC >0.125 mg/L). Conversely, the MICs of imipenem were often higher than those of ceftriaxone, regardless of the MIC of ceftriaxone (Figure 1). In ceftriaxone-resistant isolates, the MIC_50_ of ertapenem, imipenem and meropenem were 0.012 mg/L (0.004–0.032 mg/L), 0.75 mg/L (0.125–1 mg/L) and 0.032 mg/L (0.016–0.064 mg/L), respectively. The MICs of ertapenem, meropenem and imipenem were correlated with the MIC of ceftriaxone (Spearman’s ρ: 0.7120 (*P* < 0.0001), 0.6748 (*P* < 0.0001) and 0.6554 (*P* < 0.0001), respectively).

**Figure 2.**
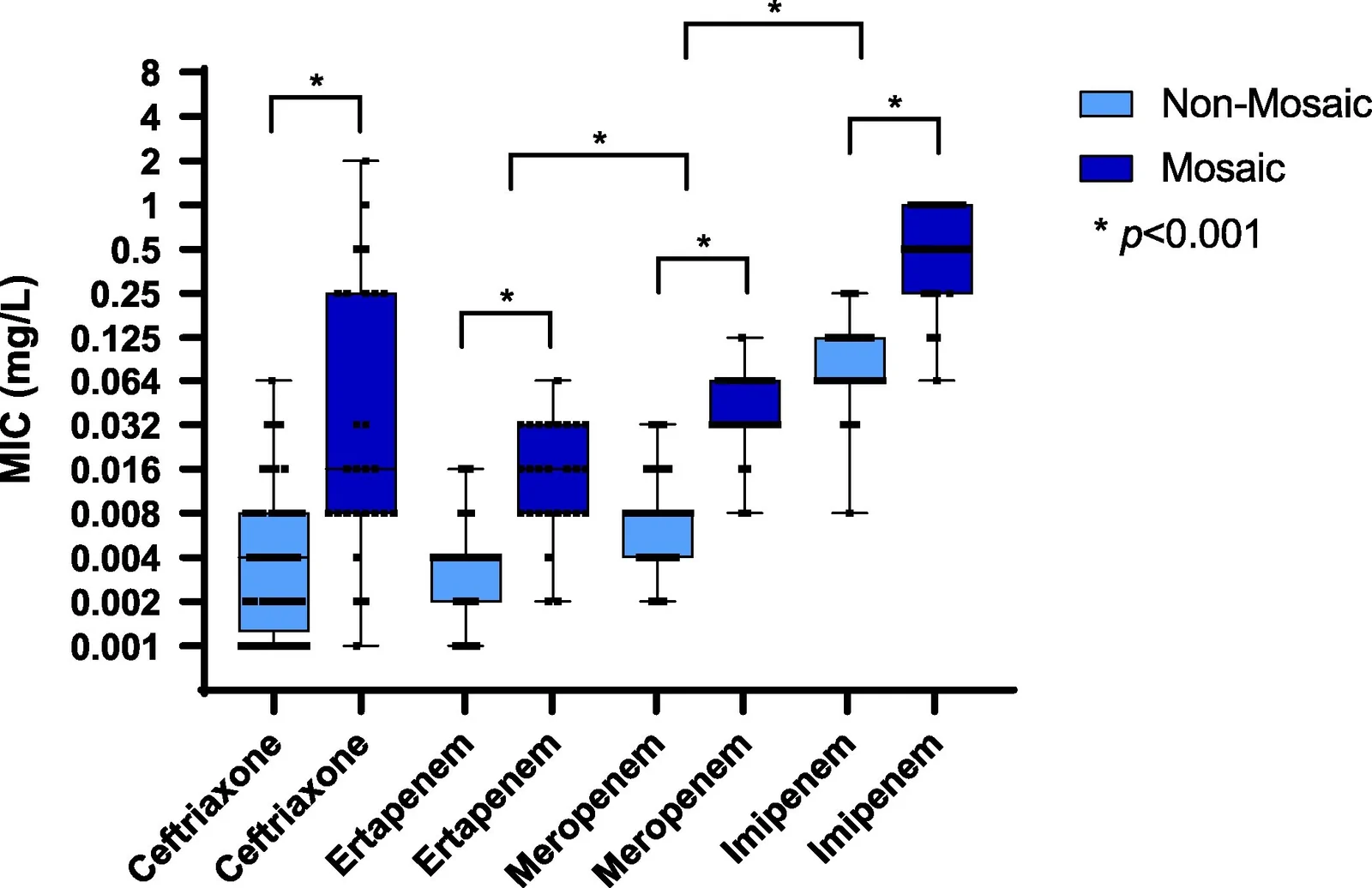
Distribution of ceftriaxone and carbapenem MICs by *penA* allele type.

The MICs of all three carbapenems were significantly higher in isolates with a mosaic *penA* allele than in those with non-mosaic alleles (*P*-value <0.001) (Figure 2). The MICs of ertapenem were significantly lower than those of meropenem, which were significantly lower than those of imipenem (*P*-value <0.001). No significant difference in carbapenem MICs was found between isolates with and without a TEM beta-lactamase (*P* = 0.65 for imipenem, *P*= 0.36 for meropenem and *P* = 0.75 for ertapenem).

To further evaluate the agreement between the two MIC determination methods, a subset of WHO reference strains was tested using both agar dilution and Etest (BioMérieux^®^). The agar dilution tests were performed using GC agar base medium supplemented with Kellogg’s supplements, according to the CLSI (formerly National Committee for Clinical Laboratory Standards) agar dilution method.^12^ Within this subgroup, the agreement between the two methods varied according to the antimicrobial agent tested. Imipenem showed the highest concordance, with 100% of MICs demonstrating essential agreement (±1 2-fold dilution). For meropenem and ceftriaxone, no MIC results differed by more than ±2 2-fold dilutions between the two methods. In contrast, ertapenem showed a lower level of concordance, with an essential agreement of 44.5%, while an additional 44.5% of MICs agreed within ±2 2-fold dilutions and the remaining 11% within ±3 2-fold dilutions (Supplementary Table S2 and Table S3).

### Conclusion and discussion

Increasing resistance to ceftriaxone may render the treatment of gonococcal infections more difficult in the future, so there is a need to identify new or known antibiotics suitable for use in first-line treatment. New oral antibiotics, such as zoliflodacin and gepotidacin, seem to be non-inferior to 500 mg ceftriaxone and 1 g azithromycin in phase III studies but have not yet been approved for market release.^13,14^ Carbapenems are extremely broad-spectrum antibiotics and are therefore not currently recommended for the treatment of uncomplicated *N. gonorrhoeae* infections caused by ceftriaxone-susceptible strains. However, in some cases of infection with XDR *N. gonorrhoeae* strains, they may be the only remaining treatment option. We show here that carbapenem MICs are generally low in *N. gonorrhoeae*, even in the presence of mosaic *penA* alleles, which increase the MICs. The presence of the *bla*_TEM-1_ or *bla*_TEM-135_ genes, which are commonly found in *N. gonorrhoeae*, encodes a narrow-spectrum β-lactamase that does not modify the MICs of carbapenems, as it does not hydrolyze them. Furthermore, no extended-spectrum β-lactamase has been reported in *N. gonorrhoeae*, supporting a lack of association between TEM production and carbapenem susceptibility.

We found differences between carbapenems, with lower MICs for ertapenem than for meropenem and imipenem. This finding is consistent with the results of a previous *in vitro* study investigating the activity of ceftriaxone and ertapenem in gonococcal isolates.^15^ This previous study investigated 274 isolates and reported similar MICs for ceftriaxone and ertapenem, except for six ceftriaxone-resistant isolates, which had a lower MIC for ertapenem than for ceftriaxone. Another study investigated the MICs of ertapenem in 62 isolates and reported that these MICs were correlated with those of cefixime and higher than those of ceftriaxone.^16^ Nevertheless, the only isolate highly resistant to cefixime and ceftriaxone had a low MIC for ertapenem, as reported in the study by Unemo *et al.*^15^ This is in line with European and Canadian guidelines, which recommend ertapenem for the treatment of cases of infection with ceftriaxone-resistant gonococcal isolates.^17^ However, there are currently no available ertapenem resistance breakpoints for *Neisseria*, and the only available carbapenem resistance breakpoint for this genus is the clinical breakpoint of 0.25 mg/L for meropenem use against *Neisseria meningitidis.*^18^ The lower concordance observed between agar dilution and Etest for ertapenem MIC determination suggests that Etest may systematically underestimate ertapenem MICs compared with the reference method. Consequently, the lower ertapenem MICs observed relative to other carbapenems when measured by Etest may be partly influenced by this methodological bias. Therefore, comparisons of ertapenem MICs with those of other carbapenems based solely on Etest results should be interpreted with caution.

Like other β-lactams, ertapenem is a time-dependent antibiotic, with a half-life of about 4 h, allowing once-daily administration. The activity of ertapenem against *N. gonorrhoeae* has not been extensively investigated *in vitro*. However, previously reported MIC_50_ and MIC_90_ values (0.008 mg/L and 0.03 mg/L, respectively) are similar to those reported for other Gram-negative bacteria susceptible to ertapenem.^19^ The MIC_50_ values obtained in our study (0.004 mg/L for the total collection and 0.012 mg/L for ceftriaxone-resistant isolates) are consistent with these findings. Notably, MICs remained low in ceftriaxone-resistant isolates, with only a modest increase relative to susceptible strains. For carbapenems, a T > MIC of approximately 20% is associated with a bacteriostatic effect, whereas a T > MIC of about 40% is required for bactericidal activity.^19^ Pharmacokinetic studies have shown that exposure is similar following the intramuscular and intravenous administration of 1 g ertapenem, with an intramuscular bioavailability of 92% and a C_max_ of 70.6 µg/mL.^20^ These data suggest that a single intramuscular administration of 1 g ertapenem would provide adequate exposure against *N. gonorrhoeae*. There is also some, albeit limited, clinical evidence supporting the activity of ertapenem against ceftriaxone-resistant *N. gonorrhoeae*. One recent case report described the successful treatment of two ceftriaxone-resistant infections with different ertapenem regimens: 1 g administered intravenously once daily for a pharyngeal infection and 1 g administered intravenously three times daily for a rectal infection.^21^ Nevertheless, uncertainties remain regarding the penetration of ertapenem at extragenital sites, particularly the pharynx, and its pharmacodynamic activity against *N. gonorrhoeae*. Additional pharmacokinetics/pharmacodynamics data, including time-kill studies and probability of target attainment analyses, are now required, together with larger clinical studies, to allow definitive conclusions to be drawn.

Only a few clinical randomized studies have evaluated ertapenem for the treatment of gonorrhoea. One randomized non-inferiority trial (NABOGO) demonstrated the non-inferiority of a single dose of 1 g ertapenem relative to a single dose of 500 mg ceftriaxone for the treatment of urogenital gonorrhoea. However, a significantly higher rate of adverse effects, mostly diarrhoea, was reported for ertapenem treatment.^22^ However, all the isolates studied were susceptible to ceftriaxone, so additional studies on ceftriaxone-resistant isolates are required, together with studies focusing on pharyngeal gonorrhoea, which is often more difficult to treat. In the UK, Fifer *et al*. described 29 ceftriaxone-resistant *N. gonorrhoeae* isolates obtained from 2015 to 2024 with ertapenem MICs ranging from 0.008 to 0.064 mg/L. In this retrospective study, ertapenem was used in three cases of pharyngeal or rectal treatment failure following the administration of 1 g ceftriaxone, and successful clearance was achieved.^9^ Ertapenem is a well-known molecule with a long history of use, and these low MICs suggest that it could be used as an alternative, even in cases of reduced susceptibility to third-generation cephalosporins. Nevertheless, breakpoints for *N. gonorrhoeae* resistance would need to be established to facilitate clinical use, and future studies involving a larger and more representative panel of isolates evaluated by the agar dilution method would provide a more robust assessment of carbapenem susceptibility in *N. gonorrhoeae.*

## Supplementary Material

dlag201_Supplementary_Data

## Supplementary data

Tables S1–S3 are available as Supplementary data at *JAC-AMR* Online.

## References

[1] Anon . Global Progress Report on HIV, Viral Hepatitis and Sexually Transmitted Infections, 2021. Accountability for the Global Health Sector Strategies 2016–2021: Actions for Impact. 1st ed. World Health Organization, 2021.

[2] Nerlander L, Champezou L, Gomes Dias J et al Sharp increase in gonorrhoea notifications among young people, EU/EEA, July 2022 to June 2023. Euro Surveill 2024; 29: 2400113. 10.2807/1560-7917.ES.2024.29.10.2400113 38456219 PMC10986672

[3] Anon . WHO bacterial priority pathogens list, 2024: Bacterial pathogens of public health importance to guide research, development and strategies to prevent and control antimicrobial resistance. https://www.who.int/publications/i/item/9789240093461. 10.1016/S1473-3099(25)00118-5 PMC12367593 40245910

[4] Ouk V, Pham CD, Wi T et al The enhanced gonococcal surveillance programme, Cambodia. Lancet Infect Dis 2023; 23: e332–3. 10.1016/S1473-3099(23)00479-6 37549683

[5] Caméléna F, Mérimèche M, Brousseau J et al Emergence of extensively drug-resistant *Neisseria gonorrhoeae*, France, 2023. Emerg Infect Dis 2024; 30: 1903–6. 10.3201/eid3009.240557 39084693 PMC11347006

[6] Golparian D, Cole MJ, Sánchez-Busó L et al Antimicrobial-resistant *Neisseria gonorrhoeae* in Europe in 2020 compared with in 2013 and 2018: a retrospective genomic surveillance study. Lancet Microbe 2024; 5: e478–88. 10.1016/S2666-5247(23)00370-1 38614111

[7] Nakayama S-I, Shimuta K, Furubayashi K-I et al New ceftriaxone- and multidrug-resistant *Neisseria gonorrhoeae* strain with a novel mosaic penA gene isolated in Japan. Antimicrob Agents Chemother 2016; 60: 4339–41. 10.1128/AAC.00504-16 27067334 PMC4914677

[8] Anon . Updated recommendations for the treatment of *Neisseria gonorrhoeae*, *Chlamydia trachomatis*, and *Treponema pallidum* and new recommendations on syphilis testing and partner services. https://www.who.int/publications/i/item/9789240090767. 39137269

[9] Fifer H, Doumith M, Rubinstein L et al Ceftriaxone-resistant *Neisseria gonorrhoeae* detected in England, 2015–24: an observational analysis. J Antimicrob Chemother 2024; 79: 3332–9. 10.1093/jac/dkae369 39417254 PMC11638718

[10] Unemo M, Ross J, Serwin AB et al 2020 European guideline for the diagnosis and treatment of gonorrhoea in adults. Int J STD AIDS 2020;0(0): 956462420949126. 10.1177/0956462420949126 33121366

[11] Maubaret C, Caméléna F, Mrimèche M et al Two cases of extensively drug-resistant (XDR) *Neisseria gonorrhoeae* infection combining ceftriaxone-resistance and high-level azithromycin resistance, France, November 2022 and May 2023. Euro Surveill 2023; 28: 2300456. 10.2807/1560-7917.ES.2023.28.37.2300456 37707979 PMC10687985

[12] Anon. Clinical and Laboratory Standards Institute (CLSI) . *Performance Standards for Antimicrobial Susceptibility Testing. 36th ed. CLSI standard M100*. Clinical and Laboratory Standards Institute. 2026. https://clsi.org/shop/standards/m100/.

[13] Ross JDC, Wilson J, Workowski KA et al Oral gepotidacin for the treatment of uncomplicated urogenital gonorrhoea (EAGLE-1): a phase 3 randomised, open-label, non-inferiority, multicentre study. Lancet 2025; 405: 1608–20. 10.1016/S0140-6736(25)00628-2 40245902

[14] Luckey A, Altarac D, McLeod SM. Letter to the editor regarding “Alternative drugs for the treatment of gonococcal infections: old and new”. *Expert Review of Anti-infective Therapy* 2024; **22**(9): 753–759. Expert Rev Anti Infect Ther 2026; 24: 647–8. 10.1080/14787210.2025.2502799 40323198

[15] Unemo M, Golparian D, Limnios A et al In vitro activity of ertapenem versus ceftriaxone against *Neisseria gonorrhoeae* isolates with highly diverse ceftriaxone MIC values and effects of ceftriaxone resistance determinants: ertapenem for treatment of gonorrhea? Antimicrob Agents Chemother 2012; 56: 3603–9. 10.1128/AAC.00326-12 22547617 PMC3393402

[16] Quaye N, Cole MJ, Ison CA. Evaluation of the activity of ertapenem against gonococcal isolates exhibiting a range of susceptibilities to cefixime. J Antimicrob Chemother 2014; 69: 1568–71. 10.1093/jac/dkt537 24492260

[17] Jensen JS, Unemo M. Antimicrobial treatment and resistance in sexually transmitted bacterial infections. Nat Rev Microbiol 2024; 22: 435–50. 10.1038/s41579-024-01023-3 38509173

[18] Anon . The European Committee on Antimicrobial Susceptibility Testing. Breakpoint tables for interpretation of MICs and zone diameters. Version 15.0, 2025. https://www.eucast.org. https://www.eucast.org/fileadmin/src/media/PDFs/EUCAST_files/Breakpoint_tables/v_15.0_Breakpoint_Tables.pdf.

[19] Zhanel GG, Wiebe R, Dilay L et al Comparative review of the carbapenems. Drugs 2007; 67: 1027–52. 10.2165/00003495-200767070-00006 17488146

[20] Musson DG, Majumdar A, Birk K et al Pharmacokinetics of intramuscularly administered ertapenem. Antimicrob Agents Chemother 2003; 47: 1732–5. 10.1128/AAC.47.5.1732-1735.2003 12709348 PMC153313

[21] Fagan L . The invisible tide of Neisseria gonorrhoeae treatment failures: a review and commentary. Clin Infect Dis 2026; 81: S223–30. 10.1093/cid/ciaf532 41504565

[22] de Vries HJC, de Laat M, Jongen VW et al Efficacy of ertapenem, gentamicin, fosfomycin, and ceftriaxone for the treatment of anogenital gonorrhoea (NABOGO): a randomised, non-inferiority trial. Lancet Infect Dis 2022; 22: 706–17. 10.1016/S1473-3099(21)00625-3 35065063

