# Supplementary material for: In vitro carbapenem activity in Neisseria gonorrhoeae
Source: JAC Antimicrob Resist. 2026 Sep 3;8(5):dlag201. doi: 10.1093/jacamr/dlag201 (PMC13539353; doi:10.1093/jacamr/dlag201)
Supplement: Supplementary file 1 [file dlag201_supplementary_data.docx]

Supplementary Table S1. Characteristics of the *N. gonorrhoeae* isolates included in the study

| **Isolate** | **MLST** | ***penA* allele** | **mosaic allele** | ***bla*TEM** | **MDR/XDR** | **Ceftriaxone MIC (mg/L)** | **Imipenem MIC (mg/L)** | **Meropenem MIC (mg/L)** | **Ertapenem MIC (mg/L)** |
| --- | --- | --- | --- | --- | --- | --- | --- | --- | --- |
| 18MONCHU9 | 1599 | 43.002 | no | Absence | non WT non MDR | 0.016 | 0.064 | 0.008 | 0.004 |
| 18TOUCHU11 | 1927 | 18.001 | no | *bla*_TEM-135_ | non WT non MDR | 0.032 | 0.25 | 0.016 | 0.008 |
| 19BORCHU11 | 1579 | 9.001 | no | Absence | non WT non MDR | 0.008 | 0.125 | 0.032 | 0.008 |
| 19CAECHU3 | 13734 | 63.001 | yes | Absence | non WT non MDR | <0.002 | 0.5 | 0.032 | 0.008 |
| 19LYICHU3 | 10314 | 2.002 | no | Absence | non WT non MDR | 0.002 | 0.125 | 0.008 | 0.004 |
| 19MONCHU21 | 1901 | 12.001 | no | Absence | non WT non MDR | 0.016 | 0.125 | 0.016 | 0.008 |
| 20ARGECH3 | 1588 | 19.001 | no | Absence | non WT non MDR | 0.008 | 0.064 | 0.008 | 0.004 |
| 20BESCHU4 | 7822 | 3.001 | no | Absence | non WT non MDR | 0.004 | 0.125 | 0.008 | 0.004 |
| 20BESPRI4 | 1594 | 1.001 | no | Absence | WT | 0.008 | 0.064 | 0.008 | 0.004 |
| 20BRAPRI10 | 8156 | 52.002 | yes | Absence | non WT non MDR | 0.016 | 1 | 0.032 | 0.008 |
| 20BRAPRI15 | 7363 | 166.001 | yes | *bla*_TEM-1B_ | non WT non MDR | 0.008 | 0.5 | 0.064 | 0.032 |
| 20CHERCH1 | 8143 | 133.001 | yes | *bla*_TEM-135_ | non WT non MDR | 0.008 | 0.25 | 0.064 | 0.016 |
| 20CNR64 | 1588 | 19.001 | no | *bla*_TEM-1B_ | non WT non MDR | 0.002 | 0.064 | 0.004 | 0.002 |
| 20HAIPRI12 | 1583 | 65.001 | semi mosaic | *bla*_TEM-1B_ | non WT non MDR | 0.002 | 0.064 | 0.008 | 0.002 |
| 20HAIPRI23 | 1583 | 2.001 | no | *bla*_TEM-135_ | non WT non MDR | 0.008 | 0.064 | 0.008 | 0.004 |
| 20LYOCHU44 | 9363 | 2.001 | no | Absence | non WT non MDR | 0.016 | 0.064 | 0.016 | 0.004 |
| 20PARSAT10 | 7363 | 166.001 | yes | *bla*_TEM-1B_ | non WT non MDR | 0.016 | 0.5 | 0.064 | 0.032 |
| 20PARSLS24 | 1583 | 2.001 | no | *bla*_TEM-135_ | non WT non MDR | 0.004 | 0.032 | 0.004 | 0.002 |
| 20PARSLS6 | 10314 | 5.002 | no | Absence | non WT non MDR | 0.008 | 0.125 | 0.008 | 0.004 |
| 20STRCHU2 | 9363 | 2.001 | no | Absence | non WT non MDR | 0.004 | 0.064 | 0.008 | 0.004 |
| 21BESPRI002 | 1599 | 14.001 | no | Absence | non WT non MDR | <0.002 | 0.064 | 0.004 | 0.002 |
| 21BESPRI007 | 8156 | 2.001 | no | Absence | WT | <0.002 | 0.064 | 0.004 | <0.002 |
| 21BONCEG007 | 11990 | 1.001 | no | Absence | WT | 0.002 | 0.064 | 0.008 | 0.004 |
| 21BRAPRI003 | 7359 | 2.002 | no | Absence | WT | 0.004 | 0.064 | 0.008 | 0.004 |
| 21BRAPRI007 | 1594 | 1.016 | no | Absence | WT | <0.002 | 0.008 | 0.002 | <0.002 |
| 21BRAPRI011 | 8135 | 15.001 | no | Absence | WT | <0.002 | 0.032 | 0.004 | <0.002 |
| 21CNR033 | 7363 | 10.001 | yes | Absence | non WT non MDR | 0.016 | 0.5 | 0.032 | 0.032 |
| 21DENCHU018 | 7822 | 5.002 | no | Absence | non WT non MDR | 0.004 | 0.125 | 0.008 | 0.004 |
| 21DENPRI037 | 7363 | 34.007 | yes | *bla*_TEM-1B_ | non WT non MDR | 0.008 | 0.5 | 0.064 | 0.016 |
| 21DENPRI041 | 13292 | 2.001 | no | Absence | non WT non MDR | 0.002 | 0.064 | 0.008 | 0.004 |
| 21HAIPRI035 | 8143 | 67.004 | no | *bla*_TEM-135_ | non WT non MDR | 0.008 | 0.25 | 0.032 | 0.016 |
| 21HAVPRI001 | 8143 | 67.004 | no | *bla*_TEM-135_ | non WT non MDR | 0.008 | 0.25 | 0.032 | 0.016 |
| 21LENSCH013 | 9362 | 2.001 | no | Absence | non WT non MDR | 0.002 | 0.125 | 0.004 | 0.004 |
| 21LENSCH015 | 11428 | 2.001 | no | Absence | non WT non MDR | <0.002 | 0.064 | 0.004 | 0.002 |
| 21LEVPRI005 | 1901 | 34.001 | yes | Absence | non WT non MDR | 0.032 | 0.5 | 0.064 | 0.016 |
| 21LEVPRI032 | 8392 | 100.006 | no | Absence | non WT non MDR | <0.002 | 0.064 | 0.004 | <0.002 |
| 21LILCHU001 | 11975 | 45.001 | no | Absence | non WT non MDR | 0.016 | 0.125 | 0.016 | 0.004 |
| 21LYOCHU028 | 17970 | 68.001 | no | Absence | non WT non MDR | 0.008 | 0.125 | 0.032 | 0.016 |
| 21LYOCHU038 | 11975 | 22.001 | no | Absence | non WT non MDR | <0.002 | 0.125 | 0.016 | 0.004 |
| 21LYOCHU046 | 7363 | 34.007 | yes | *bla*_TEM-1B_ | non WT non MDR | 0.004 | 0.5 | 0.064 | 0.016 |
| 21LYOCHU093 | 8143 | 2.002 | no | *bla*_TEM-135_ | non WT non MDR | 0.002 | 0.064 | 0.008 | 0.002 |
| 21LYOCHU110 | 1901 | 34.001 | yes | Absence | non WT non MDR | 0.008 | 1 | 0.064 | 0.032 |
| 21METPRI023 | 15964 | 103.001 | no | *bla*_TEM-1B_ | non WT non MDR | 0.032 | 0.064 | 0.008 | 0.004 |
| 21MONCHU002 | 13266 | 2.002 | no | Absence | non WT non MDR | 0.004 | 0.125 | 0.016 | 0.008 |
| 21PARBCH003 | 11516 | 2.001 | no | *bla*_TEM-1B_ | non WT non MDR | 0.002 | 0.032 | 0.004 | 0.002 |
| 21PARGHP002 | 9362 | 2.001 | no | *bla*_TEM-1B_ | non WT non MDR | 0.002 | 0.064 | 0.008 | 0.004 |
| 21PARGHP010 | 7827 | 13.001 | no | Absence | non WT non MDR | 0.064 | 0.125 | 0.016 | 0.008 |
| 21PARHPS003 | 7823 | 160.001 | no | Absence | non WT non MDR | 0.004 | 0.064 | 0.008 | 0.004 |
| 21PARHPS004 | 11864 | 2.001 | no | Absence | non WT non MDR | <0.002 | 0.064 | 0.004 | 0.002 |
| 21PARHPS007 | 8143 | 2.002 | no | Absence | non WT non MDR | 0.002 | 0.064 | 0.004 | 0.002 |
| 21PARSAT023 | 11422 | 2.001 | no | Absence | non WT non MDR | 0.004 | 0.125 | 0.008 | 0.004 |
| 21PARSLS018 | 1583 | 34.007 | yes | *bla*_TEM-1B_ | non WT non MDR | 0.008 | 0.25 | 0.032 | 0.008 |
| 21PARSLS021 | 11706 | 5.002 | no | Absence | non WT non MDR | 0.004 | 0.064 | 0.002 | <0.002 |
| 21PARSLS029 | 7827 | 13.001 | no | Absence | non WT non MDR | 0.032 | 0.125 | 0.008 | 0.004 |
| 21PARSLS031 | 10314 | 5.002 | no | Absence | non WT non MDR | 0.002 | 0.125 | 0.004 | 0.002 |
| 21PARSLS037 | 7363 | 44.001 | no | Absence | non WT non MDR | 0.008 | 0.125 | 0.008 | 0.004 |
| 21PARSLS043 | 1583 | 34.007 | yes | *bla*_TEM-1B_ | non WT non MDR | 0.008 | 0.25 | 0.032 | 0.008 |
| 21POICHU006 | 12433 | 2.006 | no | *bla*_TEM-1B_ | non WT non MDR | <0.002 | 0.064 | 0.002 | <0.002 |
| 21POIPRI007 | 10317 | 14.001 | no | *bla*_TEM-1B_ | WT | <0.002 | 0.064 | 0.004 | <0.002 |
| 21ROCPRI001 | 7363 | 34.015 | yes | *bla*_TEM-1B_ | non WT non MDR | 0.008 | 0.5 | 0.032 | 0.032 |
| 21ROCPRI015 | 1583 | 43.002 | no | Absence | non WT non MDR | 0.008 | 0.064 | 0.008 | 0.004 |
| 21SANPRI004 | 1901 | 5.002 | no | Absence | non WT non MDR | 0.008 | 0.25 | 0.016 | 0.016 |
| 21SARPRI005 | 7829 | 2.002 | no | Absence | non WT non MDR | <0.002 | 0.064 | 0.008 | 0.004 |
| 21SARPRI007 | 11516 | 2.001 | no | Absence | non WT non MDR | 0.002 | 0.064 | 0.008 | 0.004 |
| 21SDOPRI004 | 8163 | 22.001 | no | Absence | non WT non MDR | <0.002 | 0.032 | 0.002 | <0.002 |
| 21STRCHU007 | 14422 | 52.002 | yes | *bla*_TEM-1B_ | non WT non MDR | 0.002 | 0.125 | 0.008 | 0.002 |
| 21TODRCH001 | 7822 | 5.002 | no | *bla*_TEM-1B_ | non WT non MDR | 0.004 | 0.125 | 0.008 | 0.004 |
| 21TODRCH002 | 1587 | 2.002 | no | *bla*_TEM-1B_ | non WT non MDR | 0.004 | 0.064 | 0.008 | 0.004 |
| 21TOUCHU006 | 8156-like | 14.014 | no | *bla*_TEM-1B_ | non WT non MDR | <0.002 | 0.032 | 0.004 | <0.002 |
| 21TOUCHU012 | 11971 | 2.002 | no | Absence | non WT non MDR | 0.002 | 0.064 | 0.008 | 0.004 |
| 21TOUCHU016 | 1583 | 43.002 | no | *bla*_TEM-1B_ | non WT non MDR | 0.004 | 0.032 | 0.002 | <0.002 |
| 22ANGPRI015 | 7359 | 94.001 | no | Absence | WT | <0.002 | 0.25 | 0.016 | 0.004 |
| 22PARFOU015 | 7363 | 34.007 | yes | Absence | non WT non MDR | 0.016 | 0.5 | 0.032 | 0.016 |
| 22PARSAT015 | 9362 | 242.001 | no | Absence | non WT non MDR | 0.008 | 0.25 | 0.032 | 0.016 |
| 22VANCHU001 | 1580 | 93.001 | semi mosaic | Absence | non WT non MDR | 0.008 | 0.25 | 0.032 | 0.016 |
| WHOA | 10316 | 100.008 | no | Absence | WT | <0.002 | 0.032 | 0.002 | <0.002 |
| WHOC | 10931 | 156.001 | no | Absence | non WT non MDR | 0.016 | 0.125 | 0.008 | 0.008 |
| WHOD | 1902 | 12.001 | no | Absence | non WT non MDR | 0.032 | 0.125 | 0.008 | 0.004 |
| WHOG | 1903 | 2.001 | no | Absence | non WT non MDR | 0.008 | 0.125 | 0.008 | 0.004 |
| WHOK | 7363 | 10.001 | yes | Absence | non WT non MDR | 0.032 | 1 | 0.125 | 0.064 |
| WHOP | 8127 | 2.001 | no | Absence | non WT non MDR | 0.002 | 0.064 | 0.016 | 0.004 |
| F92 | 1901 | 237.001 | yes | Absence | MDR | 0.5 | 0.5 | 0.064 | 0.032 |
| F93 | 16406 | 60.001 | yes | Absence | XDR | 0.25 | 1 | 0.032 | 0.008 |
| F94 | 16406 | 60.001 | yes | Absence | XDR | 0.25 | 1 | 0.032 | 0.016 |
| WHOX = H041 | 7363 | 37.001 | yes | Absence | MDR | 1 | 1 | 0.064 | 0.032 |
| WHOY = F89 | 1901 | 42.001 | yes | Absence | MDR | 2 | 0.125 | 0.016 | 0.004 |
| WHOZ | 7363 | 64.001 | yes | Absence | MDR | 0.25 | 0.5 | 0.032 | 0.008 |
| F90 | 1903 | 60.001 | yes | Absence | MDR | 0.5 | 1 | 0.016 | 0.008 |
| F95 | 16406 | 60.001 | yes | *bla*_TEM-135_ | XDR | 0.25 | 1 | 0.064 | 0.032 |
| F96 | 16406 | 60.001 | yes | *bla*_TEM-135_ | XDR | 0.25 | 1 | 0.032 | 0.008 |

Supplementary Table S2. Concordance between MICs determined by agar dilution and Etest (BioMérieux®).

|  |  | WHO A | WHO C | WHO D | WHO G | WHO K | WHO P | WHO X | WHO Y | WHO Z |
| --- | --- | --- | --- | --- | --- | --- | --- | --- | --- | --- |
| *Ertapenem* | |  |  |  |  |  |  |  |  |  |
|  | Agar dilution (GC medium) | ≤0.002 | 0.032 | 0.016 | 0.032 | 0.125 | 0.008 | 0.125 | 0.008 | 0.032 |
|  | Etest (Biomerieux) | ≤0.002 | 0.008 | 0.004 | 0.004 | 0.064 | 0.004 | 0.032 | 0.004 | 0.008 |
|  | MIC previously published * | - | - | - | - | 0.064 | 0.004 | 0.064 | 0.008 | 0.016 |
| *Imipenem* | |  |  |  |  |  |  |  |  |  |
|  | Agar dilution (GC medium) | 0.032 | 0.125 | 0.125 | 0.125 | 0.5 | 0.125 | 1 | 0.25 | 1 |
|  | Etest (Biomerieux) | 0.032 | 0.125 | 0.125 | 0.125 | 1 | 0.064 | 1 | 0.125 | 0.5 |
| *Meropenem* | |  |  |  |  |  |  |  |  |  |
|  | Agar dilution (GC medium) | 0.008 | 0.032 | 0.032 | 0.032 | 0.064 | 0.032 | 0.125 | 0.032 | 0.064 |
|  | Etest (Biomerieux) | 0.002 | 0.008 | 0.008 | 0.008 | 0.125 | 0.016 | 0.064 | 0.016 | 0.032 |
| *Ceftriaxone* | |  |  |  |  |  |  |  |  |  |
|  | Agar dilution (GC medium) | ≤0.002 | 0.016 | 0.016 | 0.016 | 0.064 | 0.016 | 1 | 1 | 0.5 |
|  | Etest (Biomerieux) | ≤0.002 | 0.016 | 0.032 | 0.008 | 0.032 | 0.002 | 1 | 2 | 0.25 |
|  | MIC previously published * | - | - | - | - | 0.064 | 0.004 | 2 | 1 | 0.5 |

ERT: ertapenem, IMP: imipenem, MRP: meropenem, CRO: ceftriaxone

Supplementary Table S3. Essential agreement and agreement within ±2 and ±3 twofold dilutions between agar dilution and Etest (BioMérieux®) MIC results

|  | Essential Agreement  ±1 dilution (%) | Agreement within  ±2 twofold dilutions (%) | Agreement within  ±3 twofold dilutions (%) |
| --- | --- | --- | --- |
| Ertapenem | 44.5% | 44.5% | 11% |
| Imipenem | 100% | 0% | 0% |
| Meropenem | 56% | 44% | 0% |
| Ceftriaxone | 89% | 11% | 0% |

ERT: ertapenem, IMP: imipenem, MRP: meropenem, CRO: ceftriaxone
